# Utilization of Cardiac Magnetic Resonance Imaging for Assessing Myocardial Fibrosis in Prognosis Evaluation and Risk Stratification of Patients with Dilated Cardiomyopathy

**DOI:** 10.31083/RCM25654

**Published:** 2025-01-14

**Authors:** Xin-Yi Feng, Yu-Cong Zheng, Ying-Xia Yang, Wen-Feng He, Fan Yang, Ling-Li Wang, Han-Feng Yang, Chun-Ping Li, Xiao-Xue Xu, Rui Li

**Affiliations:** ^1^Department of Radiology, Affiliated Hospital of North Sichuan Medical College, 637000 Nanchong, Sichuan, China; ^2^Department of Radiology, Tsinghua University Hospital, Tsinghua University, 100084 Beijing, China; ^3^Department of Radiology, Guangxi Hospital Division of The First Affiliated Hospital, Sun Yat-sen University, 530021 Nanning, Guangxi, China; ^4^Department of Cardiology, Affiliated Hospital of North Sichuan Medical College, 637000 Nanchong, Sichuan, China

**Keywords:** cardiac magnetic resonance, dilated cardiomyopathy, myocardial fibrosis, late gadolinium enhancement, T1 mapping imaging, prognosis

## Abstract

Dilated cardiomyopathy (DCM) is the ultimate manifestation of the myocardial response to various genetic and environmental changes and is characterized mainly by impaired left ventricular systolic and diastolic function. DCM can ultimately lead to heart failure, ventricular arrhythmia (VA), and sudden cardiac death (SCD), making it a primary indication for heart transplantation. With advancements in modern medicine, several novel techniques for evaluating myocardial involvement and disease severity from diverse perspectives have been developed. Myocardial fibrosis is a significant contributor to VA events and SCD. Based on different pathological mechanisms, myocardial fibrosis can be categorized into replacement and interstitial forms. Late gadolinium enhancement (LGE) derived from cardiovascular magnetic resonance is the clinical gold standard for evaluating replacement myocardial fibrosis and exhibits high concordance with histological replacement fibrosis. However, because of the absence of normal tissue as a control, the LGE technique often fails to effectively visualize diffuse interstitial fibrosis. In such cases, T1 mapping and extracellular volume fraction mapping can be complementary or alternative methods to the LGE technique for detecting interstitial fibrosis. This review aimed to provide a comprehensive and precise assessment of myocardial fibrosis and to determine the use of cardiac magnetic resonance imaging for prognostic evaluation and risk stratification of patients with DCM.

## 1. Introduction

Dilated cardiomyopathy (DCM) affects approximately 0.04% of adults and is the most prevalent form of cardiomyopathy [1]. However, the reported 5-year mortality rate can range from 20% to 50% [2, 3], indicating significant variability in patient prognosis. This exposes some individuals to elevated risks of mortality. Therefore, accurately identifying high-risk patients and guiding their prognosis are of utmost importance.

The left ventricular ejection fraction (LVEF) remains pivotal in the current risk stratification of patients with DCM [4, 5]. However, significant LVEF reduction has not been observed in most patients with DCM who experienced sudden cardiac death (SCD) events over the past 15 years [6, 7], indicating that LVEF is an imperfect predictor of SCD. This is because the contraction and relaxation of the left ventricle involve a complex coordinated process which is described by the “ventricular myocardial band theory” [8, 9]. In the early stages of the disease, the myocardium compensates to maintain LVEF, making it less reliable for detecting subtle changes [10, 11]. However, LVEF measurement is more valuable for evaluating patients with advanced heart failure (HF) because, at this stage, the compensatory capacity of the myocardium may have become depleted and a more pronounced decrease in LVEF can be observed.

Research indicates that approximately one-third of deaths among patients with DCM may be caused by SCD or ventricular arrhythmia (VA) events [12, 13]. As a critical complication of DCM, SCD events refer to the rapid occurrence of unexpected death or cardiac arrest caused by cardiovascular causes, which occur outside of the hospital or in the emergency room, whereas ventricular fibrillation resulting from ventricular tachycardia is the most common mechanism leading to SCD events [14]. Myocardial fibrosis, characterized by replacement, interstitial, and perivascular fibrosis, constitutes a pivotal histological foundation for arrhythmias [15]. Numerous studies have convincingly reported that replacement or interstitial fibrosis alters cardiac electrophysiology by impeding the propagation rate of action potential, augmenting ectopic automaticity, initiating re-entrant rhythm, and facilitating after-depolarization, thereby triggering the aforementioned arrhythmias [16, 17, 18].

Late gadolinium enhancement (LGE) based on cardiovascular magnetic resonance (CMR) is a noninvasive imaging technique that enables multiplane and multiparameter imaging for qualitative and quantitative evaluation of replacement myocardial fibrosis. The working principle of this technique lies in the selective accumulation of gadolinium in the extracellular space, whereas necrosis or fibrotic areas increase extracellular volume, prolonging the clearance time for the contrast agent within these regions [19]. Consequently, when imaging is performed at a specific time after the clearance of the contrast agent from the healthy myocardium, enhanced features are observed in fibrotic areas due to their non clearance [20].

Although observed in approximately 30%–40% of patients with DCM [21, 22], observational data have shown that patients with LGE have a very high mortality rate, which has been identified as a strong and independent predictor of SCD, VA, cardiac mortality, and all-cause mortality [23]. Therefore, assessing patients for LGE, including comprehensive analysis of its extent, location, and pattern, is a critical determinant of prognosis in DCM because this information has recently received significant attention as an approach for enhancing our understanding of the implications of LGE positivity [24].

However, in approximately 60% of patients with DCM, LGE negativity on CMR should not be considered indicative of the absence of myocardial fibrosis [25]. Previous studies have demonstrated that interstitial myocardial fibrosis may indeed occur at an early stage of DCM, even in the absence of LGE [26, 27]. This form of fibrosis, characterized by the accumulation of collagen fibers in the interstitial spaces of the myocardium, can precede the development of more severe, irreversible fibrotic changes [28]. Therefore, early detection and treatment of interstitial fibrosis are crucial for preventing disease progression and improving outcome [29].

The native T1 value of the myocardium can be visually analyzed using the T1 mapping technique, which enables direct measurement of each voxel’s T1 value. Furthermore, by assessing pre- and post-enhancement myocardial and blood T1 relaxation times and calculating the extracellular volume fraction (ECV), evaluation of interstitial fibrosis within the extracellular matrix becomes possible [30]. Studies have shown a strong correlation between native T1 values (which refer to the time constant known as longitudinal relaxation time, commonly used to assess tissue water content, structural characteristics, etc.), ECV, and histological manifestations of extracellular space [26, 27]. These parameters reliably differentiate diffuse fibrotic myocardium from healthy tissues, thereby reducing the reliance on endocardial biopsy and serving as a valuable complement to LGE.

This study comprehensively reviewed recent research advancements in the LGE and T1 mapping techniques, with the aim of providing valuable insights into identifying high-risk patients and deploying early intervention strategies.

## 2. Correlation of Replacement Fibrosis with Risk Stratification and Prognosis

### 2.1 Presence of LGE

Approximately one-third of patients with DCM exhibit replacement myocardial fibrosis [31], and numerous previous studies have consistently shown a correlation between replacement myocardial fibrosis and various adverse outcomes, highlighting its independent predictive value for adverse events, such as cardiac events, all-cause mortality, and arrhythmic events (e.g., SCD, ventricular fibrillation, ventricular tachycardia) [32, 33]. The LGE technique can effectively distinguish between normal and fibrotic myocardia, making it the gold standard for the noninvasive assessment of replacement myocardial fibrosis widely employed in clinical practice [34].

Several studies have shown that the presence of LGE is a robust prognostic indicator of adverse outcomes in patients with DCM, particularly in terms of SCD events [7, 35, 36]. Moreover, Di Marco *et al*. [36, 37] observed that LGE exhibited superior predictive efficacy for arrhythmia events, particularly in patients without significant LVEF decline (hazard ratio [HR]: 10.4; *p* < 0.001). This is a particularly noteworthy finding because it challenges the traditional reliance on LVEF as the primary indicator of risk in patients with DCM. The superior predictive power of LGE in this context suggests that LGE imaging can identify high-risk patients who may not be captured by LVEF alone.

In patients with significant LVEF decline, the left ventricle undergoes substantial remodeling, resulting in tissue instability at a critical level. The cascade of acute electrical instability and mechanical failure can ultimately lead to SCD [38]. However, in cases where the left ventricle is not extensively remodeled, myocardial fibrosis may be a primary contributor to VA and SCD. Reduced normal cardiomyocytes within fibrotic areas lead to altered electrophysiological properties, typically characterized by low weighted unipolar voltages. These changes affect myocardial conduction and excitability, increasing the risk of VA and SCD. Additionally, the altered electrical activity may be incongruous with the original neural innervation, potentially further influencing the occurrence of VA and SCD [39].

Since the initial study in 2006 that evaluated the prognostic significance of LGE in DCM, several investigations on LGE in DCM have been conducted [40, 41]. These studies generally corroborated that LGE can serve as an indicator of adverse outcomes [42, 43, 44]. However, the predictive capability of LGE across different studies has been substantially inconsistent, particularly concerning various clinical endpoints [45, 46, 47]. In particular, this variability may manifest as a HR of 1.45 for composite endpoint events and reach as high as 14 for arrhythmic events [34, 48]. These observations suggest that relying on LGE alone is not sufficient to achieve the desired accuracy of risk stratification. Therefore, incorporating more detailed LGE-related indicators is crucial for enhancing the effectiveness of LGE in predicting adverse outcomes. These refined indicators will enable a more accurate assessment of patient condition and risk stratification, facilitating the implementation of precise risk stratification strategies. (The brief summary of the cited article is shown in Table 1 (Ref. [6, 7, 20, 25, 26, 31, 34, 35, 36, 37, 40, 41, 42, 43, 44, 45, 47, 48, 49, 50, 51, 52, 53, 54, 55, 56, 57, 58, 59])).

**Table 1.** **The brief summary of the cited article in the review**.

First author (Ref. #)		Year	Type of study	Patients enrolled	Endpoint
Presence of LGE					
	Gulati [7]	2013	Prospective	472	Primary end point (all-cause mortality), secondary end points (cardiovascular mortality or cardiac transplantation; an arrhythmic composite of SCD or aborted SCD; and a composite of HF death, HF hospitalization, or cardiac transplantation).
	Becker [31]	2018	Meta-Analysis	4554	Cardiovascular mortality, major ventricular arrhythmic events (appropriate ICD therapy, rehospitalization for HF, and left ventricular reverse remodeling).
	Alba [34]	2020	Retrospective	1672	Composite primary end point (all-cause mortality, heart transplantation, or left ventricular assist device implant), secondary arrhythmic end point (SCD or appropriate ICD shock).
	Halliday [35]	2019	Prospective	874	All-cause mortality and SCD.
	Di Marco [36]	2021	Retrospective	1165	Combined arrhythmic endpoint (appropriate ICD therapies, sustained VT, resuscitated cardiac arrest, and SCD).
	Di Marco [37]	2017	Meta-Analysis	2948	Arrhythmic endpoint (sustained VA, appropriate ICD therapy, or SCD).
	Assomull [40]	2006	Prospective	101	Primary combined end point (all-cause death and hospitalization for a cardiovascular event), secondary outcome (SCD or VT).
	Klem [41]	2021	Prospective	1020	All-cause and cardiac death, SCD.
	Buss [42]	2015	Prospective	210	Composite endpoint: cardiac events together with the occurrence of hospitalization due to congestive HF.
	Lehrke [43]	2011	Prospective	184	Composite endpoint: cardiac death, hospitalization for decompensated HF, or appropriate ICD discharge.
	Yamada [44]	2014	Prospective	57	Composite endpoint: cardiac death, hospitalization for decompensated HF, or documented lethal arrhythmia, including VT and VF.
	Perazzolo [45]	2014	Prospective	137	Arrhythmic events: SCD, cardiac arrest due to VF, sustained VT, or appropriate ICD intervention.
	Tateishi [47]	2015	Prospective	207	Composite endpoint: cardiac death, cardiac transplantation, LV assist device implantation, appropriate ICD discharge for VT or VF, and rehospitalization for HF.
	Neilan [48]	2013	Prospective	162	Composite endpoint: cardiovascular death and appropriate ICD therapy; arrhythmic events: ATP, ICD discharge, and non-heart failure cardiovascular death.
Extent of LGE					
	Li [6]	2023	Retrospective	466	Primary end point (SCD or aborted SCD), secondary end point (all-cause mortality, heart transplant, or hospitalization for HF).
	Barison [25]	2020	Prospective	183	Composite endpoint: appropriate ICD shock and cardiac death; arrhythmic events: appropriate ICD shock.
	Halliday [35]	2019	Prospective	874	All-cause mortality and SCD.
	Di Marco [36]	2021	Retrospective	1165	Combined arrhythmic endpoint (appropriate ICD therapies, sustained VT, resuscitated cardiac arrest, and SCD).
	Puntmann [49]	2016	Prospective	637	All-cause mortality.
	Li [50]	2022	Retrospective	659	Primary endpoints (cardiac-related death and heart transplantation). Secondary endpoints (hospitalization for HF, VA, and ICD or cardiac resynchronization therapy implantation).
	Romano [51]	2018	Prospective	1012	All-cause death.
	Behera [52]	2020	Retrospective	112	Composite endpoint: all-cause mortality, resuscitated cardiac arrest, sustained VT/appropriate ICD shock, HF hospitalization.
LGE locations					
	Claver [20]	2023	Retrospective	1165	Primary endpoint (appropriate defibrillator therapies, sustained VT, resuscitated cardiac arrest, or sudden death), secondary outcome (HF hospitalizations, heart transplant, left ventricular assist device implantation, and end-stage HF death).
	Barison [25]	2020	Prospective	183	Composite endpoint: appropriate ICD shock and cardiac death; arrhythmic events: appropriate ICD shock.
	Halliday [35]	2019	Prospective	874	All-cause mortality and SCD.
	Di Marco [36]	2021	Retrospective	1165	Combined arrhythmic endpoint (appropriate ICD therapies, sustained VT, resuscitated cardiac arrest, and SCD).
	Behera [52]	2020	Retrospective	112	Composite endpoint: all-cause mortality, resuscitated cardiac arrest, sustained VT/appropriate ICD shock, HF hospitalization.
	Xu [53]	2021	Prospective	412	Composite endpoint: all-cause mortality and HF readmission; all-cause mortality.
LGE patterns					
	Alba [34]	2020	Retrospective	1672	Composite primary end point (all-cause mortality, heart transplantation, or left ventricular assist device implant), secondary arrhythmic end point (SCD or appropriate ICD shock).
	Halliday [35]	2019	Prospective	874	All-cause mortality and SCD.
	Di Marco [36]	2021	Retrospective	1165	Combined arrhythmic endpoint (appropriate ICD therapies, sustained VT, resuscitated cardiac arrest, and SCD).
	Behera [52]	2020	Retrospective	112	Composite endpoint: all-cause mortality, resuscitated cardiac arrest, sustained VT/appropriate ICD shock, HF hospitalization.
	Xu [53]	2021	Prospective	412	Composite endpoint: all-cause mortality and HF readmission; all-cause mortality.
	Gulati [54]	2019	Prospective	100	Not applicable.
	Li [55]	2022	Retrospective	39	Cardiac death or transplant, and more major adverse cardiovascular events.
Integrated LVEF strata with LGE status					
	Li [6]	2023	Retrospective	466	Primary endpoint (SCD or aborted SCD), secondary end point (all-cause mortality, heart transplant, or hospitalization for HF).
	Di Marco [36]	2021	Retrospective	1165	Combined arrhythmic endpoint (appropriate ICD therapies, sustained VT, resuscitated cardiac arrest, and SCD).
T1 and ECV mapping					
	Nakamori [26]	2018	Retrospective	36	Not applicable.
	Li [50]	2022	Retrospective	659	Primary endpoints (cardiac-related death and heart transplantation). Secondary end points (hospitalization for HF, VA, and ICD or cardiac resynchronization therapy implantation).
	Kitagawa [56]	2022	Retrospective	45	Combined cardiac events (cardiac death, VT/VF, HF hospitalization).
	Nakamori [57]	2020	Prospective	115	Primary endpoint (composite of appropriate ICD therapy and SCD).
	Vita [58]	2019	Prospective	241	Major adverse cardiac events (HF hospitalizations and deaths).
	Li [59]	2023	Prospective	858	SCD-related events (SCD, appropriate ICD shock, and resuscitation after cardiac arrest).

**Note**: LGE, late gadolinium enhancement; VT, ventricular tachycardia; VF, ventricular fibrillation; HF, heart failure; ICD, implantable cardioverter-defibrillator; SCD, sudden cardiac death; LV, left ventricular; LVEF, left ventricular ejection fraction. ATP, anti-tachycardia pacing; ECV, extracellular volume fraction; VA, ventricular arrhythmias.

### 2.2 Extent of LGE

Considering the high prevalence of LGE, reliance on the presence of LGE only as an indication of adverse events may not be sufficient. The multiplane and multiparameter characteristics of CMR enable quantitative evaluate LGE extent. Several studies have explored the prognostic value of the extent of LGE, revealing that each 1% increase in LGE extent can be a predictor of adverse outcomes [25, 49, 50]. Furthermore, a study focusing on patients with LVEF <50% reported that each 1% increase in LGE extent was notably correlated with a heightened risk of mortality by approximately 3% [51]. Therefore, this correlation may stem from an enhanced susceptibility to re-entrant arrhythmias as the extent of LGE increases [25], highlighting the importance of the accurate quantification of LGE using CMR.

However, Halliday *et al*. [35] and Behera *et al*. [52] noted a nonlinear relationship between LGE extent and prognosis. They revealed that the risk of mortality increases with LGE extent; however, this increase was not monotonic. For instance, the HRs for SCD events were found to be 1.59 for the 0%–2.55% LGE extent group, 1.56 for the 2.55%–5.10% group, and 2.31 for the >5.10% group [35]. Despite the increase in risk with increasing LGE extent, the increase rate is not uniform, and the increase rate of HRs for all-cause mortality varies. Consequently, modeling LGE extent as a linear predictor may underestimate the risk in most patients and overestimate the risk in those with the most extensive LGE. Notably, in Halliday *et al*.’s study [35], the LGE extent that was most predictive of adverse outcomes was found to be 1.29%, with a corresponding C-statistic of 0.70.

Furthermore, a recent study revealed the interaction between LGE extent and LVEF for predicting outcomes. In particular, the study revealed that in patients with LVEF ≥35%, the predictive value of LGE for adverse events becomes particularly pronounced when LGE extent exceeds 7.1%. In these patients, the risk of SCD or aborted SCD was 4.4 times higher (HR = 4.4; 95% CI: 2.4–8.3; *p* < 0.001) [6]. This finding suggests that the combination of LGE extent and LVEF provides a more nuanced approach to risk stratification, emphasizing the importance of identifying the specific threshold of LGE extent that correlates with increased risk.

However, the current method for measuring LGE extent using CMR primarily reflects the percentage of the LGE area that occupies the left ventricular myocardial mass without considering the heterogeneity of regional LGE. The limitations of this method may lead to inaccurate assessment of cardiac structure and function. Research suggests that elements influencing the nonlinear relationship between LGE extent and prognosis include the fact that the critical isthmus sites of VA are located in tissue heterogeneity zones, which lie between myocardial fibrosis and healthy tissue [60]. Additionally, as the spatial extent and degree of heterogeneity of LGE increase, the heterogeneity of electrical conduction within cardiac tissue also rises, making it more likely for reentrant circuits to form and thereby increasing the probability of inducing arrhythmias. It is also important to note that the timing of arrhythmia induction is primarily determined by the level of local maximum fibrosis. This is because the activation patterns of arrhythmias exhibit a high degree of periodicity and regularity, and the regions with the highest degree of fibrosis will dictate the interval between arrhythmia episodes [61]. Moreover, previous studies investigating the impact of LGE extent did not adequately consider the potential effects of different LGE patterns. Di Marco *et al*. [36] found that epicardial and transmural LGE were significantly associated with a higher proportion of adverse outcomes than mid-wall LGE. However, whether epicardial and transmural LGE still maintain their high-risk characteristics at the same extent of LGE lesions remains to be clarified through further research.

The aforementioned studies have indicated that placing greater emphasis on the composition of LGE indicators rather than its volume is essential. However, there is currently a lack of consensus on the measurement methods and parameters for quantifying LGE, necessitating urgent standardization of LGE quantification methods before their use as decision-making tools. A brief summary of the cited article is shown in Table 1 (Ref. [6, 25, 35, 36, 49, 50, 51, 52]).

### 2.3 LGE Locations

The localization of LGE in patients with idiopathic DCM predominantly occurs in the septum caused by specific pathological mechanisms, whereas LGE in DCM caused by viral myocarditis is predominantly observed in the free wall [62]. This disparity may be because cardiotropic viruses causing viral myocarditis mainly enter through the bloodstream, leading to direct exposure of the free wall to inflammatory factors present in the blood due to its contact with the pericardium [63]. The microstructural characteristics of fibrosis may differ depending on DCM etiology, thereby influencing its prognostic significance.

Previous studies have primarily focused on septal LGE and identified associations between all-cause mortality, VA events, and composite endpoints [25, 35, 52, 53]. With the advancement and maturation of CMR technology, attention has gradually shifted toward LGE in other myocardial regions. Some investigations have focused on LGE within the free wall; however, there remains a debate regarding whether free wall LGE is a protective or detrimental factor, with conflicting findings from these studies (HR: 0.77 and HR: 5.42) [35, 53]. This ambiguity highlights the need for further research to delineate the clinical implications of free wall LGE. Furthermore, studies exploring LGE in the inferior wall of the free wall have uniformly revealed a notable link between inferior wall LGE and adverse outcomes [25, 52], reinforcing the importance of considering the specific LGE location in the context of cardiac disease management.

Although the prognostic assessment of isolated free wall LGE in patients with DCM remains questionable, the presence of free wall and septal LGE often indicates poor prognosis. Previous studies have investigated the impact of LGE at different locations on patient prognosis and found a significant correlation between the multiple locations (both septal and free wall) of LGE and adverse outcomes in patients with DCM [35, 36, 53]. Particularly in Halliday *et al*.’s research [35], the HR for SCD events reached 5.82 when LGE was present in both the septum and free wall. This finding supports the hypothesis that prognosis deteriorates when LGE occurs at multiple locations within the same patient due to the potential indication of greater extent and higher LGE heterogeneity.

In a recent multicenter cohort study involving 1165 consecutive patients with DCM [20], the presence of LGE at right ventricular insertion points was associated with comparable outcomes to LGE-negative patients, while significantly reducing the risk of VA and sudden death compared with cases with left ventricular LGE. When analyzing LGE, particularly when quantifying LGE extent, distinguishing between LGE at the right ventricular insertion point and LGE at other locations in the left ventricle is advisable. The brief summary of the cited article is shown in Table 1 (Ref. [20, 25, 35, 36, 52, 53]).

### 2.4 LGE Patterns

**Fig. 1.**
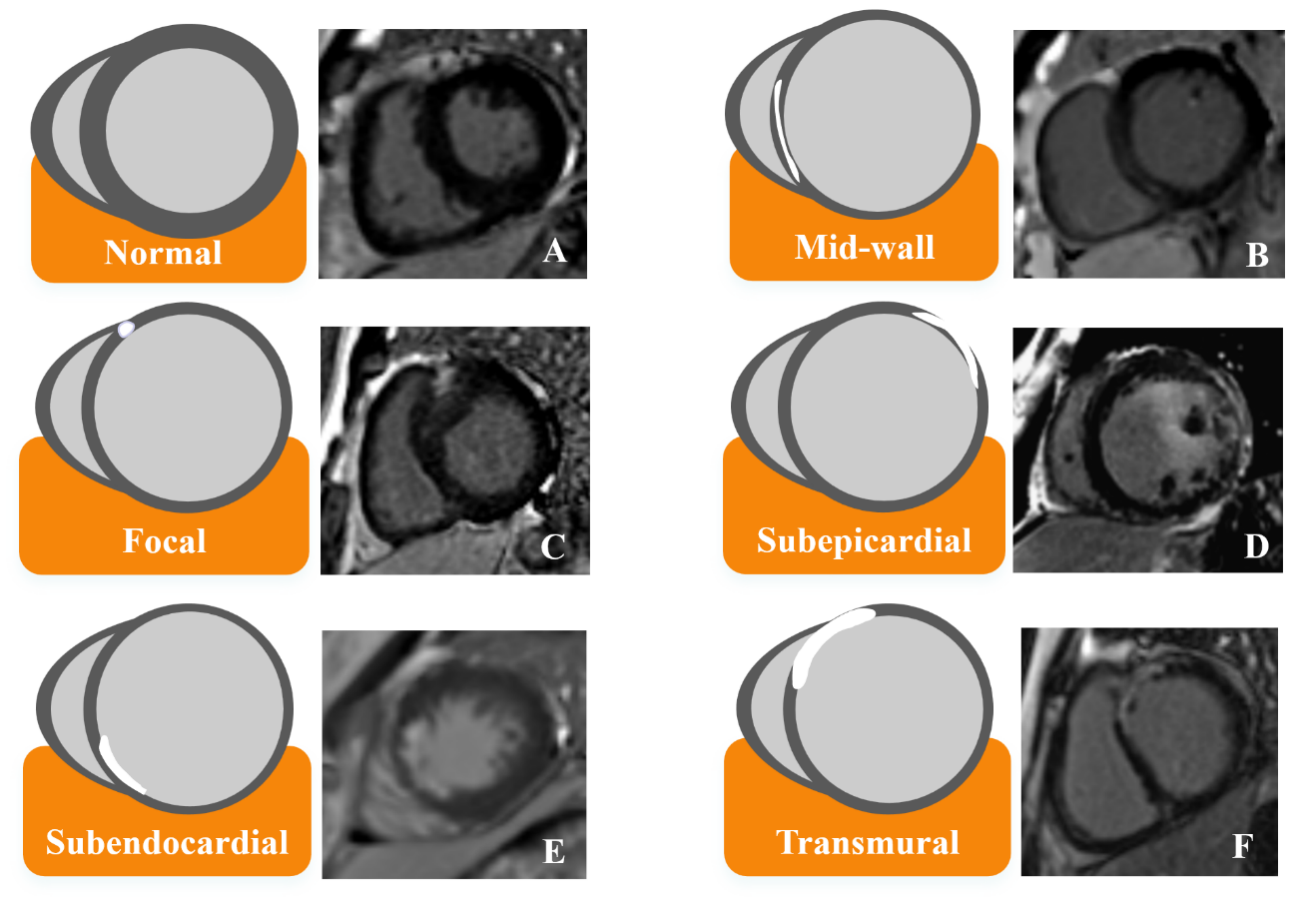
**Schematic illustration of different LGE patterns and corresponding MRI images**. (A) Normal heart. (B) The mid-wall pattern of LGE located in septum. (C) The focal pattern of LGE located in right ventricular insertion point. (D) The subepicardial pattern of LGE located in free wall. (E) The subendocardial pattern of LGE located in septum. (F) The transmural pattern of LGE located in both septum and free wall. LGE, late gadolinium enhancement; MRI, magnetic resonance imaging.

The mid-wall pattern, which is the most common manifestation of LGE, has gained widespread recognition. Concurrently, subepicardial, subendocardial, and transmural LGE patterns have also been research spotlights, drawing considerable attention (all LGE patterns are shown in Fig. 1). In particular, numerous studies have definitively highlighted subepicardial myocardial fibrosis as a key LGE pattern that indicates poor prognosis, making this a significant area of focus [35, 52, 53]. Furthermore, de Frutos *et al*. [64] demonstrated that the distribution of LGE patterns in DCM is gene-specific. In particular, LGE-negative patients exhibited a higher frequency of mutations in the *TNNT2*, *RBM20*, and *MYH7* genes, whereas those with subepicardial LGE demonstrated a greater prevalence of *DMD*, *DSP*, and *FLNC* gene mutations. These findings enable effective screening of high-risk families and provision of prognostic guidance.

Previous studies have demonstrated that subendocardial and transmural LGE patterns indicate previous myocardial infarction [7, 65]. However, some studies have identified subendocardial and transmural LGE patterns that are inconsistent with the distribution of coronary arteries and have confirmed their predictive value for adverse outcomes [34, 36]. These findings imply that these two LGE patterns cannot be simply classified as ischemic lesions. Although the etiology remains unclear and most patients with these LGE types are associated with microcirculatory disturbances and abnormal myocardial perfusion [54], it is also plausible that viral infections and other nonischemic mechanisms contribute to these patterns [55]. Consequently, it is essential to direct more attention to these neglected LGE subtypes.

However, current research shows there is controversy regarding whether the focal LGE pattern serves as a protective or adverse factor because their HR values were 3.16 and 0.21, respectively [35, 53]. This discrepancy may be attributed to the limited number of patients exhibiting this specific pattern. Furthermore, due to the thin myocardial walls in patients with DCM, distinguishing focal LGE from other patterns, such as subepicardial and subendocardial LGE, is challenging.

Similar to LGE location, diverse LGE patterns can be observed within the same patient, each exhibiting variations in fibrosis structure. The literature shows that the presence of multiple LGE patterns (the combination of mid-wall, subepicardial, and focal) is correlated with worse outcomes [34, 35, 53]. Note that Alba *et al*. [34] demonstrated a gradient response in the coexistence of multiple LGE patterns, with an elevated risk that increases as the number of patterns present increases. This trend was manifested with a HR of 1.24 with 1 pattern, 2.07 with 2 patterns, and 4.76 with >2 patterns, indicating a progressive correlation between the complexity of LGE patterns and the risk of adverse outcomes. The brief summary of the cited article is shown in Table 1 (Ref. [34, 35, 36, 52, 53, 54, 55]).

### 2.5 Integrated LVEF Strata with LGE Status

Furthermore, recent studies have integrated LVEF strata with LGE status, which has significantly enhanced the accuracy of risk stratification in patients with DCM, providing incremental valuable insights for the improvement of risk stratification [6, 36]. Di Marco *et al*. [36] identified high-risk LGE in patients with DCM, including epicardial, transmural, and combined septal and free wall LGE. Considering the differences in annual event rates among patients with different LVEF categories, the study divided LVEF values into three strata (≤20%, 21%–35%, and >35%). Combining LGE characteristics with LVEF strata, the researchers developed a risk model for patients with DCM and classified these patients into seven risk categories. Based on this model, patients with negative LGE and an LVEF >35% exhibited the lowest risk of SCD and VA events, with an annual event rate of 0.07%. In contrast, patients presenting with LGE and an LVEF <20% encounter a notably increased risk, with an annual event rate of 8.1%.

Building upon these findings, the subsequent step involves subdividing each parameter (i.e., extent, location, and pattern) of LGE and integrating them with specific cardiac function information (i.e., LVEF and myocardial deformation) or clinical indicators to potentially refine risk stratification. The brief summary of the cited article is shown in Table 1 (Ref. [6, 36]).

### 2.6 T1 and ECV Mapping

In a normal myocardium, the extracellular volume comprises intravascular compartments containing blood, and the stroma of the myocardium contains signaling molecules, such as fibrillary collagen and proteoglycans. However, in patients with cardiomyopathy, the presence of extracellular or interstitial edema, replacement fibrosis, or invasive fibrosis can increase interstitial component levels. This subsequently increases extracellular volume, prolongs myocardial T1 relaxation time, and is associated with native T1 values [30].

Native T1 values, which do not require the administration of contrast agents during imaging examinations, can serve as a complementary or alternative method for assessing LGE, particularly for patients with severe renal insufficiency or those who cannot tolerate contrast-enhanced examinations. However, technical factors, such as magnetic field strength, variations in T1 mapping techniques, and non-resonant artifacts, can introduce errors when assessing native T1 values. Furthermore, most cardiomyopathies exhibit relatively subtle changes in native T1 values, which may pose challenges for detection. ECV, representing the proportion of extracellular stromal volume in the entire myocardium, is calculated based on pre- and post-enhancement myocardial T1 values. It reflects changes in extracellular stroma and is strongly associated with extracellular matrix, as confirmed by biopsy [26]. This technique is a noninvasive method for evaluating structural changes and provides a deeper understanding of the underlying pathology of DCM. The combination of native T1 values and ECV allows for a comprehensive evaluation of interstitial myocardial fibrosis in patients with DCM. Although current research is limited, the T1 and ECV mapping techniques have shown promising prospects for prognostic evaluation of DCM.

Kitagawa *et al*. [56] demonstrated native T1 values as a predictor of left ventricular reverse remodeling and cardiac events in DCM patients. This finding is consistent with the results of another study that found native T1 values to be as predictive of arrhythmia events [57]. Complementing this, research has shown the ability of the ECV to forecast outcomes, such as HF in DCM [58]. Furthermore, a separate study demonstrated that native T1 and ECV could predict cardiac mortality and heart transplantation in patients with DCM without LGE [50].

In Li’s group [50], native T1 >936 ms and ECV >25.9% were confirmed to be independent predictors of adverse outcomes. Two other studies have discovered that per 10-ms increase in native T1 and per 3% increase in ECV were correlated with SCD events, and the number of abnormal ECV locations was also found to be linearly related to the annual major adverse cardiac events rate [58, 59]. Therefore, although T1 and ECV mapping show great promise for predicting the prognosis of patients with DCM, the evaluation metrics in each study varied, and the standardized threshold values for the relevant parameters were not determined. Furthermore, the optimal critical values for identifying high-risk individuals may differ according to different patient populations and imaging platforms. These are all issues related to T1 and ECV mapping that require attention. To address these issues, further studies using larger datasets and different patient populations are required.

The latest research has revealed that compared with using LGE or LVEF alone, models that combine LGE with native T1 or ECV are more effective in stratifying the risk of SCD in patients with DCM, highlighting a superior predictive ability [59]. Therefore, exploring the predictive value of T1 and ECV mapping in combination with other imaging markers, such as LGE and LVEF, along with further sublevel indicators, including the location and pattern of LGE, could improve risk stratification and provide a more comprehensive assessment of the underlying cardiac pathology. This multifaceted approach can help clinicians identify high-risk patients more accurately and tailor personalized management strategies to improve outcomes in this challenging patient population. The brief summary of the cited article is shown in Table 1 (Ref. [26, 50, 56, 57, 58, 59]).

## 3. Conclusions

Echocardiography and CMR can assess DCM; however, echocardiography is more commonly employed in clinical practice because of its simplicity and low cost. However, CMR offers a more precise measurement of atrial and ventricular volumes, enabling a more accurate calculation of the ejection fraction and assessment of cardiac function [66]. A previous study demonstrated that in the same population, CMR measurements yielded higher left ventricular volumes and a lower LVEF compared to echocardiography [67]. Moreover, the CMR-based model for predicting major adverse cardiovascular events outperformed the echocardiography-based model in their study [67]. Therefore, using CMR to identify subgroups of patients with echocardiography-LVEF near the threshold can help prevent the missing indications of implantable cardioverter-defibrillator.

Furthermore, the histological features obtained by CMR are crucial for patient prognosis. Detected fibrosis is persistent and dynamic, with some patients developing new fibrosis or seeing existing fibrosis progress. Therefore, echocardiography alone may miss high-risk patients, and patients should be followed, regardless of the presence of LGE on the CMR [68].

LVEF remains a key parameter in HF treatment, despite not being an optimal predictor of SCD [69]. It helps to categorize patients into different subgroups for tailored treatments, significantly improving the prognosis for patients. Furthermore, combining LVEF with LGE enhances predictive capabilities. By integrating LVEF with other markers, its predictive power can be enhanced even further [6, 36].

The LGE, T1 mapping, and ECV mapping techniques derived from CMR exhibited significant prognostic value in predicting adverse outcomes among patients with DCM. Furthermore, this study performed in-depth comparisons and analyzed the emerging indicators of LGE (e.g., extent, location, and pattern) and reviewed recent research advancements in the integrated application of myocardial fibrosis-related risk indicators, providing a novel perspective for optimizing the risk stratification model. Note that no comprehensive studies have focused on the assessment of perivascular fibrosis, indicating a significant potential for further exploration in this field. Furthermore, studies have shown that Sodium-Glucose Linked Transporter 2 inhibitors not only treat diabetes but also inhibit cardiac fibrosis, and research is needed to see if CMR can detect their effects on myocardial fibrosis, potentially aiding in drug therapy assessment [70, 71].
